# Assessment and Measurement of the Side-Effects of an Evidence-Based Intervention with an Advanced Smart Cricket Ball Exemplified by a Case Report on Correcting Illegal Bowling Action

**DOI:** 10.3390/s26010299

**Published:** 2026-01-02

**Authors:** René E. D. Ferdinands, Batdelger Doljin, Franz Konstantin Fuss

**Affiliations:** 1 Faculty of Medicine and Health, University of Sydney, Sydney, NSW 2141, Australia; 2 Smart Products Engineering Program, Swinburne University, Melbourne, VIC 3000, Australia; batdelger.doljin@gmail.com; 3 Chair of Biomechanics, Faculty of Engineering Science, University of Bayreuth, D-95447 Bayreuth, Germany; franzkonstantin.fuss@uni-bayreuth.de; 4 Division of Biomechatronics, Fraunhofer Institute for Manufacturing Engineering and Automation IPA, D-95447 Bayreuth, Germany

**Keywords:** ball sports, spin bowling, performance parameters, spin rate, torque, precession, power, acceleration, efficiency, centre of pressure

## Abstract

Correcting an illegal bowling action in cricket through technical intervention involves modifying a movement pattern. This study evaluated the success of an intervention aimed at correcting the suspect actions of two off-spin bowlers and assessed whether it influenced other movement patterns and performance parameters that were not directly targeted by the intervention (the “side-effects” of the intervention). For this purpose, a smart cricket ball equipped with three high-speed gyroscopes was used. This technology not only detects illegal bowling actions but also measures five physical and five skill performance parameters, along with ratios within and across these two parameter groups. Additionally, it tracks the position of the centre of pressure, where torque is applied to the ball, and identifies the type of delivery based on the direction of the angular velocity vector in the global coordinate system. The significant reduction of 15–27% in the integral of the precession signal before the torque spike provided the required evidence for the success of the intervention. Among the performance parameters not targeted by the intervention, skill parameters improved by 7–53% after the intervention relative to before the intervention, while physical performance parameters (spin rate, torque, angular acceleration) decreased (≈10%) significantly after the intervention. The position and vector of the centre of pressure shifted significantly distally and closer to the palm in both bowlers (33.3° and 47.5° in participants A and B, respectively) after the intervention. Although the basic average type of delivery (sidespin plus half swerve, and top-spin plus half swerve) did not change, the yaw angle of the angular velocity vector in the global coordinate system moved significantly counterclockwise by 18–20°. This study demonstrated that while the intervention successfully corrected illegal bowling actions, the smart cricket ball also revealed changes in other performance measures, which aligns with the understanding that the bowling action operates as an integrated, dynamic system.

## 1. Introduction

Before prescribing effective bowling techniques to improve performance outcomes within the legal constraints of the bowling laws, the process must begin by understanding the biomechanical principles that govern bowling actions and analysing the dynamics of the bowling arm. Cricket bowling was traditionally considered a rigid-arm motion with no elbow straightening during delivery [1]. However, research indicates that many bowlers do not maintain a perfectly rigid arm during the action, convincing administrators to modify the rules to allow an elbow extension limit, which corresponds to observed bowling mechanics in practice [2].

In response to the concerns about illegal bowling actions, researchers have applied biomechanical models to gain deeper insights into the relationship between elbow mechanics and performance outcomes in bowling. For instance, the manipulation of additional elbow flexion in a forward kinematic model of the bowling arm resulted in increased wrist velocity [3]. In a simplified two-link model of the bowling arm, elbow flexion increases the radius of rotation from the humerus to the wrist, thereby enhancing the contribution of humeral internal rotation to ball speed [4]. To apply this mechanism in bowling, the elbow typically undergoes a period of extension during the arm-acceleration phase, while leaving sufficient flexion at release to allow humeral rotation to contribute to ball velocity, a feature that is observable in baseball pitching and the tennis serve [5]. However, there is a tendency for suspect bowlers to overextend their elbows, promoting an action with throw-like qualities.

In cricket, the rules limit elbow extension between the arm reaching horizontal and ball release to 15 degrees [6], specifically to prevent bowlers from indirectly exploiting a throw-like mechanism to generate high levels of humeral internal rotation. Research suggests that elbow extension or hyperextension during ball release does not increase bowling speed and can even reduce it [3]. Hence, the purpose of this legal constraint is to limit a bowler’s ability to increase ball speed via humeral internal rotation, helping to preserve the unique mechanical characteristics of bowling. Exceeding the 15-degree limit [7] could provide an unfair advantage in terms of ball speed or spin, emphasising the importance of strict rule enforcement to maintain the integrity of the sport [8]. It should be noted that the 15° elbow extension limit does not prohibit elbow hyperextension. A bowler who hyperextends the elbow and then recoils optimally by flexing the elbow during ball release, thereby increasing ball speed, may be able to exploit a legal mechanical advantage [9].

There are several methods for assessing illegal bowling. Traditionally, match umpires have relied solely on visual inspection, without the aid of technical assistance. However, the accuracy and feasibility of making such judgments from the umpire’s position and perspective have been questioned [10]. To assess illegal bowling actions outdoors without access to a motion analysis lab, coaches typically use high-frame-rate cameras, including modern smartphones, to film the bowler from two or more planar perspectives. Video footage can be analysed frame-by-frame using applications such as Hudl Technique or Coach’s Eye to assess elbow extension relative to the ICC’s 15-degree limit and to identify technical flaws such as misalignment or irregular arm paths.

The gold standard for evaluating the legality of bowling actions is 3-D motion analysis, in which participants perform trials with markers on the major joints of their bowling arm [2,3,4,11,12,13,14]. Outdoor options are also available, such as markerless camera systems [15] or wearable technology [16,17,18,19,20,21,22]. In the first case, Portus et al. [7] used two synchronised high-speed video cameras operating at 250 Hz and digitised the bowlers’ joint centres in each frame. Although this method is accurate (error ±1°), the camera installation was dependent on the existing infrastructure and digitisation time-consuming. Wearable technology [16,17,18,19,20,21,22] utilises inertial measurement units (IMUs), which contain MEMS gyroscopes and accelerometers, to measure the angular velocity and linear acceleration of the upper arm and forearm during cricket bowling. Although MEMS gyroscopes have high accuracy in planar movements, 3D movements with additional rotations compromise the accuracy if their coordinate system is not precisely aligned with the flexion-extension axis of the elbow joint. A more accurate approach involves electrogoniometers, which directly measure the elbow joint’s flexion–extension angle [23,24]. The disadvantage of the wearable technology solutions is that they need to be attached to the bowler’s arm, e.g., with tight-fitting fabric sleeves, which could affect the bowling performance.

An alternative method is to use the advanced smart cricket ball, which is a novel technology that supports both the assessment of bowling legality and the coaching intervention process [25,26]. Equipped with state-of-the-art sensors and analytical capabilities, this device captures detailed kinematic data while retaining the appearance and feel of a conventional cricket ball. The smart ball measures both the spin rate (angular velocity of the ball in the hand and during flight) and the speed of movement of the angular velocity vector across the ball’s surface, known as precession. In other words, when the applied torque is not perfectly aligned with the intended spin axis, the spin axis drifts during the release phase. This makes precession a direct indicator of off-axis torque application during finger–ball interaction, independent of any aerodynamic effects once the ball is in flight. For coaches, precession can be understood more simply as a “wobble” of the spin axis at release, indicating that some of the effort is being “wasted” rather than fully transferred into the intended spin.

From the sports engineering perspective, precession is characterised by increased peak values and a greater area under the precession signal, arising from torques that are not parallel to the angular velocity vector [27]. Critically, elevated levels of precession have been linked with excessive elbow extension immediately before ball release [28], a finding that has been validated against gold-standard motion-analysis systems capable of identifying elbow extension beyond the ICC’s 15° threshold [6]. Such research validates the smart ball design objectives, namely, to deliver real-time feedback and detailed performance analysis, while retaining the appearance and feel of a conventional ball. This approach enables the smart ball to be integrated into practice sessions and facilitates tasks such as delivery identification [29], thereby providing data that can directly inform coaching intervention measures, such as correcting illegal bowling actions.

The ICC’s (International Cricket Council) legal procedures for handling doubtful actions provide bowlers who exceed the elbow extension limit with the opportunity to undergo coaching intervention programs [6]. If suspect action mechanics are identified, targeted drills like shadow bowling, resistance band exercises, and simplified bowling actions are designed to correct the technique, emphasising proper arm motion, body alignment, and follow-through [30]. Previous findings suggest that corrective interventions combining resistance work and simplified action-based drills have empirical support for improving biomechanical performance in bowling or other athletic motions [30,31,32]. Feedback provided through mirrors or video playback enhances coaching effectiveness, while re-testing with the same filming setup enables monitoring of progress and compliance with legal bowling standards. This combined intervention approach may increase the likelihood of remediating illegal bowling actions.

Unfortunately, there is still a pressing need for scientific validation of coaching interventions that are prescribed to remediate illegal bowling actions. Ramalho and Petrica [33] highlight that the efficacy of these interventions depends on meticulous biomechanical analysis and empirical evidence. Moreover, biomechanical processes demonstrate that segment movements are intricately linked, and dependent on interactions between segments [34,35]. This means that an intervention may target change in one performance parameter, but inadvertently affect others, positively or negatively, highlighting the challenge of evaluating the effectiveness of schemes to modify existing movement sequences.

The purpose of this study was to assess the advanced smart cricket ball’s effectiveness in validating interventions related to correct illegal bowling actions, where the term “validation” refers to whether the intervention was actually carried out, regardless of whether it was performed correctly or whether it was successful. By evaluating a wide range of analytical parameters, this research aims to bridge the gap between biomechanical theory and practical coaching applications, promoting evidence-based coaching practices based on empirical validation. Through collaboration among biomechanists, engineers, coaches, and players, this study seeks to advance the understanding of cricket bowling mechanics while promoting the integrity and fairness of the sport.

The specific objectives of this study were:

(1) To evaluate a cricket coaching process holistically using an advanced smart cricket ball, drawing on standard performance parameters provided by the device, specifically focusing on physical and skill parameters, centre of pressure position, type of spin-bowling delivery, and detection of throwing motion.
(2) To detect the “side-effects” of an intervention, i.e., how the intervention influences other movement patterns and performance parameters that were not directly targeted by the intervention, specifically whether side-effects can be detected at all, how can they be measured and quantified, and which parameters are affected in the first place, by comparing performance parameters before and after the intervention.
(3) To assess the suitability of the smart cricket ball for documenting coaching interventions based on its performance data (including target parameters and side effects), irrespective of the intervention’s success.
(4) To apply a coaching process to bowlers with suspected illegal actions, with a particular focus on identifying and analysing the side effects of the intervention.

## 2. Materials and Methods

### 2.1. The Advanced Smart Cricket Ball

The smart cricket ball used in this study was developed in late 2011 (mark 1 [25]) and its wireless version (mark 2) was built in 2014 [26]. The ball records the angular velocity *ω* with three orthogonally aligned single-axis high-speed gyros with a sampling frequency of 815 Hz, wirelessly transmits the data to a laptop or smartphone, and charges via induction. The electronics are miniaturised and have a maximum diameter of 29 mm. From the angular velocity, the software (Smart Cricket Ball Analysis, version 21, proprietary software of F. K. Fuss and B. Doljin) calculates 4 further physical and 5 skill performance parameters [27]: the torques imparted on the ball (resultant *T_R_*, spin torque *T_S_*, and precession torque *T_P_*); the angular acceleration *α*; the power *P*; the precession *p* (speed of the movement of the **ω**-vector, caused by *T_P_*); the normalised precession *p_n_* (angle *θ* between the **ω**-vector and the **T_R_**-vector); the efficiency *η* (ratio of actual to ideal angular kinetic energy, if *θ* and thus *T_P_* were zero); and the ‘frequency’ (ratio of *α*_max_ to release *ω*_max_). Note that for a sine wave, the latter skill parameter *α*_max_/*ω*_max_ would be 2π*f*, where *f* denotes the frequency of the sine wave [27].

### 2.2. Participants

Two amateur off-spin bowlers (Bowler A and Bowler B) were recruited for testing with the smart cricket ball and video analysis. Off-spin bowling is a type of spin delivery in cricket where a right-hand bowler rotates the fingers in a clockwise sense to impart spin on the ball [13,36]). Relative to a right-hand batter, such a ball turns from the off-side (the side of the batter’s chest during stance) to the leg-side (the side closer to the batter’s legs) after it bounces on the pitch. In other words, the spin is such that the ball deviates from left to right (from the bowler’s perspective) after hitting the ground, increasing the challenge of the batter to respond with an appropriate stroke. The major inclusion criteria were: (i) off-spin as their primary bowling style and (ii) absence of upper-limb injury at the time of testing.

This research received Ethics approval from the Human Ethics Committee of Swinburne University (approval no. 20191582-3216, 2019) and ethically followed the Declaration of Helsinki.

### 2.3. Qualitative Legality Assessment

We developed a qualitative assessment tool for evaluating the legality of bowling actions, drawing on established biomechanical literature related to suspect bowling actions [2,8,13,24], and further informed by the mechanics of overarm throwing as examined in baseball research [37,38]. Given that illegal bowling actions involve excessive elbow extension, it is biomechanically reasonable to expect that other throwing-related characteristics, such as those observed in baseball pitching, may also be present in suspect deliveries. Accordingly, the assessment instrument included 14 parameters spanning five domains: elbow kinematics, shoulder and scapular control, arm trajectory, trunk posture, and stride mechanics.

Central to the tool were indicators derived from throwing biomechanics. For instance, maximum shoulder external rotation (MER) and elbow flexion during the cocking and acceleration phases have been identified as critical determinants of joint loading and energy transfer in baseball pitchers [37,38]. These variables were included to capture possible compensatory mechanisms in suspect bowling actions and were assigned higher weightings due to their strong biomechanical relevance. Poor control of scapulothoracic motion, particularly insufficient scapular retraction or elevation during arm cocking in pitching, can further amplify joint stress [39,40] and could plausibly increase the likelihood of elbow extension violations during the arm-acceleration phase.

Importantly, this scapular movement does not occur in isolation, since it is typically accompanied by lumbar extension, which allows the thorax to tilt posteriorly and accommodates the extreme arm-cocking position required for powerful ball release. The greatest lumbar extension occurring at the point of maximum shoulder external rotation has been observed in a large sample of baseball pitchers [41]. Therefore, the inclusion of a combined scapulothoracic motion ± lumbar extension variable serves as a valid indicator of possible throwing mechanics and enhances the discriminatory power of the legality assessment tool.

Another throwing-related parameter integrated into the tool was excessive contralateral trunk flexion during arm-acceleration, often observed in high-velocity throwing sports to facilitate momentum transfer. While it may contribute to ball speed, it also increases trunk and shoulder loading and is considered a deviation from classical fast bowling technique. Oyama et al. [42] highlighted the link between lateral trunk tilt (bending) and increased upper limb stress in baseball pitching, thus justifying its inclusion as an indirect marker of a “throwing-type” action in cricket.

The assessment also incorporated trunk orientation parameters, notably front-on alignment at back foot contact, based on evidence that bowlers reported for illegal actions frequently exhibited this positioning in the pelvis and thorax [13]. This alignment can compromise optimal kinetic sequencing and force redirection, potentially predisposing athletes to compensatory arm actions. Additionally, we included minimal trunk flexion at release and a reduced V-angle (the angle between the front thigh and the torso) as markers of reduced biomechanical efficiency. A sufficiently large V-angle reflects effective use of ground reaction forces and is associated with legal, energy-efficient bowling mechanics [43]. In contrast, limited trunk flexion and a shallow V-angle may result in reduced power transfer from the lower body to the upper limb, increasing reliance on the arm and thereby raising the likelihood of illegal motion.

Lower-body contributions were assessed through stride length and stride alignment, both of which play critical roles in the efficient transmission of ground reaction forces (GRFs) during the bowling action. Shorter strides or excessively open stride angles relative to the target line can disrupt kinetic sequencing and reduce the bowler’s ability to effectively harness and redirect GRFs. Research by King et al. [44] found that a larger horizontal (braking) impulse is associated with higher ball speeds and a greater front-leg plant angle, which is biomechanically linked to stride length. This suggests that optimal stride mechanics can maximise performance while maintaining legal technique.

In terms of developing an assessment variable, stride length and plant angle must be interpreted relative to the bowler’s anthropometrics. For instance, athletes with shorter stature or narrower shoulder width may naturally present with proportionally longer plant angles for the same absolute stride length. Given the general correlation between shoulder width and height, we used stride length normalised to shoulder width as a practical index for biomechanical comparison. This standardisation accounts for individual variability and provides a more meaningful interpretation of stride-related mechanics.

Importantly, stride characteristics also influence trunk orientation at ball release, particularly the V-angle (the angle between the trunk and lead thigh; [43]). Reduced stride length or suboptimal stride alignment can compromise trunk positioning and energy transfer up the kinetic chain, reinforcing the inclusion of these variables to design an integrated biomechanical assessment framework.

Finally, two variables were included based on visual or qualitative signs traditionally associated with throwing. The first was a jerky or whipping arm motion, which was a historical criterion for suspect actions in the Laws of Cricket prior to their revision in 2000. Although no longer codified, it remains a practical observational cue used by umpires and coaches. The second was the vertical orientation of the bowling arm at release, which, when exaggerated (i.e., “above-the-vertical” technique), may reflect poor segmental sequencing or overcompensation in arm trajectory, and is frequently observed in suspect deliveries.

The 14 biomechanical parameters of the participants were assessed by a professional cricket coach using both naked eye and high-speed video recordings captured on a mobile phone. Each participant’s deliveries were recorded from multiple angles to ensure visibility of the critical joints and body segments. The coach examined the video frame-by-frame, focusing on key phases of the bowling action, particularly the arm-acceleration phase, ball release, and back foot contact.

For each parameter, the coach determined the presence and magnitude of the movement based on visual inspection and expert judgment:

1. Excessive elbow extension during arm-acceleration—evaluation of straightening of the elbow from horizontal arm position to ball release.
2. Jerky or whipping motion at release—appearance of abrupt changes in arm velocity or acceleration.
3. Excessive shoulder external rotation (ER)—observation of shoulder rotation exceeding the typical anatomical range for bowlers at the horizontal arm position.
4. Scapular-thoracic motion ± lumbar extension—assessment of upper back and shoulder blade movements, including compensatory lumbar extension.
5. Visible elbow flexion at maximal shoulder ER—inspection of elbow bending at peak shoulder external rotation.
6. Early shoulder abduction—premature lateral movement of the upper arm due to the unsupported “falling-away” of the action.
7. Bowling arm above vertical at release—observation of arm position relative to vertical at the moment of release.
8. Release point significantly ahead of front foot—evaluation of ball release relative to front foot position.
9. Minimal trunk flexion at release or head well ahead of the front foot at release—assessment of forward bending of the torso and the relative head position.
10. Minimal “V-angle” (trunk–front thigh)—inspection of the angular relationship between torso and lead thigh.
11. Front-on alignment at back foot contact—evaluation of the orientation of the torso and shoulders when the back foot contact.
12. Excessive lateral bending ± shoulder rotation—observation of side bending or rotation beyond normal range.
13. Open stride angle ± open foot position—assessment of lead foot orientation and stride width.
14. Stride length < 1.2 × shoulder width (short)—assessment of step length noticeably short, in an unathletic position.

Each assessment item was scored on a 0–4 scale, estimating the level of elbow extension during the bowling action from visual observation of slow-motion video footage. Each rating examined a critical feature directly or indirectly associated with elevated levels of elbow extension. A professional coach with experience in assessing suspect actions (REDF) rated each item for Participants 1 and 2. The rating uses a 5-point scale, commonly adopted in qualitative observational analysis. The correspondence between the ratings and legality evaluation is shown below:

0—No observable change in elbow angle.

1—Trace or inconsistent change, well below 15°.

2—Mild, visible change estimated within the legal tolerance (~10–15°).

3—Moderate straightening judged to be approaching or slightly exceeding 15° (~15–20°), likely to trigger formal testing.

4—Marked appearance of straightening beyond 15°, qualifying as suspect, and most likely illegal.

Each score was multiplied by a predetermined weight (Wt) ranging from 1 to 3 (Table 1 and Table 2), based on its biomechanical relevance and potential impact on legality. For example, items related to elbow extension and elbow flexion at MER were weighted most heavily (Wt = 3) due to their strong association with illegal actions. The maximum composite score was 76, with higher totals indicating a greater number or severity of biomechanical violations. The idea was to create a structured yet flexible evaluation of bowling legality, capturing both quantitative movement deviations and qualitative visual features.

### 2.4. Bowling Trials

Each participant bowled 12 deliveries (equivalent to two overs) for an initial assessment. During this phase, no coaching feedback was provided. Bowling actions were evaluated using both qualitative visual assessment by a professional coach (REDF) (Table 1) and objective measurement via a smart cricket ball equipped with three high-speed gyroscopes. The qualitative assessment identified both participants as having “suspect” actions, characterised by the appearance of excessive elbow extension during the critical arm-acceleration phase.

### 2.5. Intervention Trials

**Figure 1:**
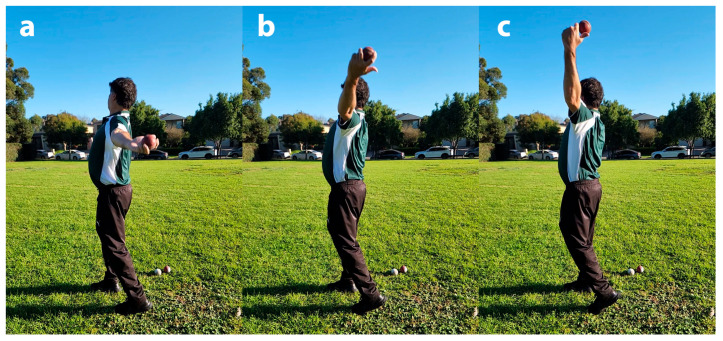
Intervention method for correcting illegal bowling action; (**a**) The intervention begins with the bowler using less shoulder external rotation and a relatively extended elbow; (**b**) as the shoulder externally rotates, the bowler focuses on rapidly supinating the forearm and flexing the elbow during the upswing; (**c**) the flexing wrist and middle finger, working synergistically with elbow flexion, helps the bowler achieve a legal bowling action.

A 30-min corrective intervention of the bowling techniques, grounded in biomechanical and motor control principles, was implemented by the coach (REDF) (Figure 1). The session began with detailed verbal instructions and demonstrations, highlighting the target positions and key cues for proper elbow and arm motion. Bowlers then engaged in a series of guided practice trials, which included shadow bowling, slow-motion rehearsal, and partial deliveries to reinforce the correct movement patterns [30,31,32]. Continuous corrective feedback, both verbal and visual, was provided to the bowlers. The number of trials was tailored to each bowler’s performance, and practice continued until the coach observed consistent execution of the targeted patterns within the 30-min timeframe.

From a technical perspective, the coaching protocol targeted excessive elbow extension during the critical arm-acceleration phase, from the horizontal extension of the bowling arm behind the bowler to the point of ball release, which is the period for assessing legality [6]. While direct research on the synergy between elbow and wrist flexion in healthy individuals is limited, ref. [44] observed that wrist flexor activation (flexor carpi radialis) occurs simultaneously with elbow flexion, whereas wrist extensor activation (extensor carpi ulnaris) accompanies elbow extension. Hence, the intervention focused on developing neuromuscular control and coordination between wrist flexion, forearm supination, and elbow flexion, to promote legally compliant movement patterns while preserving the natural rhythm and timing of the bowling action. The objective was to reduce the likelihood of illegal elbow extension caused by habitual or dynamic coupling, which extends to the shoulder joint [45,46].

A further critical aspect of the intervention was ensuring that the bowlers limit shoulder external rotation and maintain a relatively extended elbow when the bowling arm was approximately horizontal behind the back (Figure 1a). Once this characteristic was achieved consistently, the focus shifted to rapid forearm supination and elbow flexion during the upswing above horizontal (Figure 1b). This adjustment exploits the biomechanical principle that maximum wrist flexion torque occurs in forearm supination [47]; Figure 1c), creating a natural synergistic effect. To enhance this synergy, bowlers were also instructed to flex the fingers toward the wrist joint during ball release, further facilitating wrist flexion and promoting coordinated movement patterns [48]. We hypothesised that coordinating these movements would appreciably reduce elbow extension, appreciably reducing extension, with the possibility of even inducing elbow flexion during the release phase, thereby facilitating the objective of remediating the suspect off-spin bowling actions.

Although spin bowlers generate lower ball speeds than fast bowlers, a greater proportion of their energy is transferred into frictional finger torque to generate spin [49]. Consequently, spin bowling is an athletic motion that mirrors the fundamental energy-generating mechanics of fast bowling, albeit on a smaller scale. Hence, similar to fast bowlers, spin bowlers cannot achieve optimal efficiency if their stride length is insufficient to fully apply the braking impulse generated by the front foot [50]. Without this impulse, the transfer of momentum through the kinetic chain is reduced, forcing the elbow to compensate mechanically to achieve the desired performance. In this intervention, maintaining a stable base with an appropriately sized stride is fundamental for spin bowlers to enhance arm control and minimise compensatory movements that could contribute to excessive elbow extension during the release phase.

### 2.6. Data Analysis

**Figure 2:**
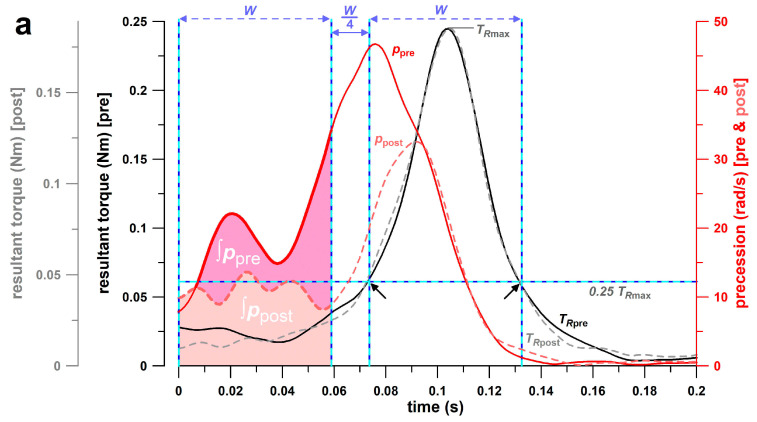

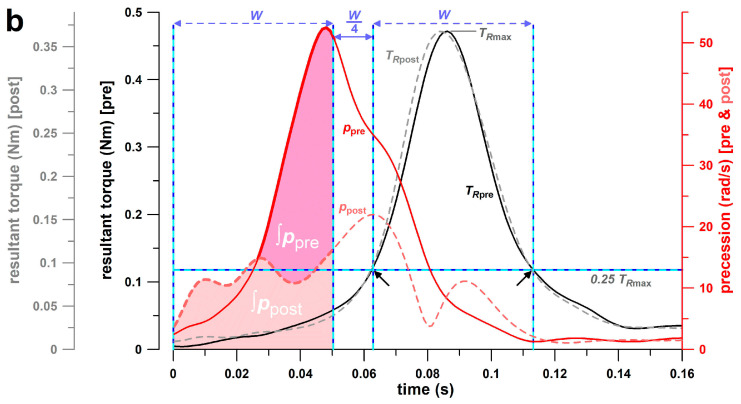
Examples of torque and precession data vs time before and after the intervention of (**a**) participant A and (**b**) participant B; *T_R_*, resultant torque; *p*, precession; *W*, width of time window at ¼ of maximum *T_R_* (black single headed arrows); ∫*p*, integral of precession *p* across a time window of width *W*; pre & post: data before & after the intervention; continuous lines: before intervention; dashed lines: after intervention.

While the effect of the intervention training was assessed visually during and after the intervention study, the evidence of success or failure of the intervention training was based on a novel parameter (∫*p*) developed specifically for the assessment of illegal bowling [28] by the authors of this study, where the first author (REDF) executed the legal and illegal off-spin deliveries. The rationale behind this method was that extending the elbow (chucking, throwing) before releasing the ball adds another torque component to the ball’s spin that is not aligned to the future spin axis of the released ball. For example, in fast bowling, the motion of the straight arm before releasing the ball imparts a topspin torque on the ball, while the ball is released with backspin. Consequently, the spin axis (angular velocity vector **ω**) must change direction by almost 180°. The speed of the moving **ω**-vector equals the precession *p* [27]. The parameter ∫*p* was used to quantify the angular displacement of the angular velocity vector (ω) relative to the ball’s coordinate system during the release phase. Lower *p*_max_ and ∫*p* values reflect reduced secondary torques acting on the ball, resulting in more stable orientation of the ω-vector during release. A larger ∫*p* indicates greater elbow flexion angles and faster elbow extension speeds [28]. To calculate ∫*p*, the following procedure was applied. First, the time window W was identified as the period during which the release torque (TR) exceeded 25% of its maximum value (TRmax). This window was then shifted earlier in time by 1.25 × *W* to capture the pre-release motion of the ball (Figure 2). Finally, the precession signal (*p*) was integrated across this adjusted time window to obtain ∫*p*, representing the total angular displacement of the ω-vector within that phase of the delivery.

**Figure 3:**
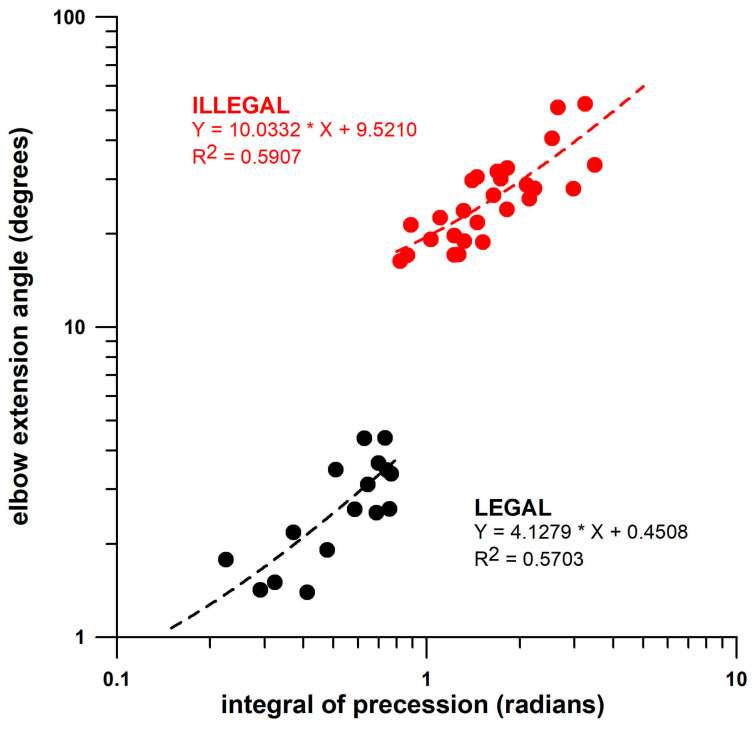
Elbow extension angle (degrees) vs ∫*p* (radians), the integral of precession *p* (cf. Figure 2); black dots: legal deliveries; red dots: illegal deliveries [28].

**Figure 4:**
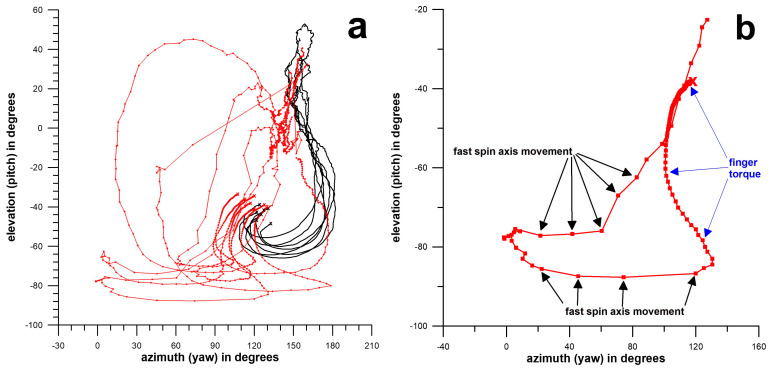
Elevation vs azimuth angles of the angular velocity vectors (**ω**) moving on a path curve over the surface of the smart ball (black curves: legal fingerspin sidespin; red curves: illegal fingerspin sidespin); x = release points; (**a**): 7 legal and 7 illegal deliveries; (**b**) path curve of illegal delivery showing the zones of high precession (fast movement of the **ω**-vector caused by elbow extension) and zone where the fingers impart the torque on the ball [28].

**Figure 5:**
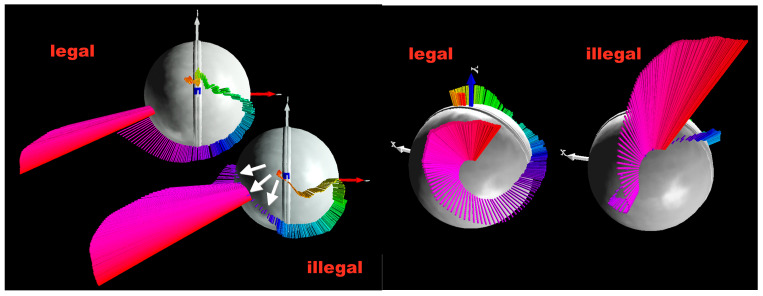
Vector diagrams of the angular velocity (**ω**) shown on the surface of the smart ball from two different views (legal and illegal deliveries of a fingerspin sidespin); The three white arrows mark the part of the **ω**-vectors that have different positions in illegal bowling compared to legal bowling; the **ω**-vectors are colour-coded where the colours represent the time in rainbow colours (red-orange-yellow-green-blue-purple-magenta-red) [28].

The data from the previous study [28] included 16 legal and 27 illegal deliveries from the same bowler, including fingerspin and wristspin (backspin, backsidespin, sidespin, topsidespin, and topspin). The integral of precession *p* (∫*p*) was determined using the smart cricket ball, and the elbow angle was measured using a Cortex motion analysis system with markers at the shoulder, elbow, and wrist (Figure 3). The medians of the ∫*p* data (below and above 15°) differed highly significantly (*p* < 0.0001, effect size r = 1). The data shown in Figure 3 are expected to depend on the specific movement patterns of individual bowlers and may therefore not be comparable between different bowlers. However, the data can be compared within the same bowler with respect to improvement after interventions. Figure 4 and Figure 5 illustrate that legal and illegal deliveries are based on fundamentally different movements. Figure 4 shows the pitch and yaw angles of the axis of rotation (angular velocity vector **ω**) relative to the ball’s coordinate system. While legal deliveries exhibit a clockwise rotation of the axis of rotation, illegal deliveries show a predominantly counterclockwise movement before a final clockwise movement where the fingers finally impart torque on the ball. The three-dimensional representation of Figure 4 is shown in Figure 5, where the actual **ω**-vectors are depicted on the ball’s surface. In illegal bowling, the vectors on the negative (−z) hemisphere of the ball move closer to the y- and x-axes of the ball’s coordinate system [28].

Side-effects were defined as unintended changes in bowling performance parameters not directly targeted by the corrective intervention, including but not limited to: peak precession, normalised precession, centre-of-pressure coordinates, yaw and pitch angles of the angular velocity vector, spin rate (ω), maximum spin torque (*T_S_*), maximum angular acceleration (*α*_max_), and derived ratios such as *T_S__*_max_/*T_P__*_max_, *α*_max_/*ω*_max_, and ω/*T_R__*_max_. These parameters were analysed to identify any secondary effects of the intervention on ball-release mechanics in three ways:

(a) Ten performance parameters (5 physical and 5 skill) were calculated for each ball played before and after the intervention, using the smart ball software [27] (Smart Cricket Ball Analysis, version 21, proprietary software of F. K. Fuss and B. Doljin).
(b) The centre of pressure, which represents the point of torque application, was calculated according to the protocol of Fuss et al. [51]. For comparison purposes, the bowling hand was aligned with the ball’s coordinate system (BCS), the index finger was placed on the x-axis (seam plane = xy-plane), and the z-axis (pole of the ball) pointed into the palm of the right hand.
(c) The delivery type was identified by the smart ball after aligning the BCS with the global coordinate system (GCS; x—forward, direction of the ball’s flight; y—to the left; z—up) [29]. To achieve this alignment, the BCS was continuously rotated about the instantaneous angular velocity **ω**-vector by the angle *φ*, i.e., by the magnitude of the instantaneous *ω*/*f*, where *f* is the data sampling frequency (815 Hz). After each completed rotation step, the rotated BCS was rotated back into its original and initial position along with the instantaneous **ω**-vector [29]. Because the participants were right-handed off-spinners, the yaw (azimuth) and pitch (elevation) angles of the **ω**-vector in the GCS were both expected to be at 0° ± 22.5°, if the off-spin was a perfect side-spin.

### 2.7. Statistics

The data of all balls delivered, 24 per participant, were divided into two groups, namely bowling before and after the intervention. The medians and interquartile ranges were calculated for each group and each performance parameter. Pre- and post-intervention values were compared using a Mann–Whitney U test to detect significant differences (*p* < 0.05) and to determine the effect size *r* (*r* = 1 − 2U n_1_^−^ n_2_^−1^). This non-parametric approach was chosen because of the very small sample size and the likelihood that the data would not meet the normality assumptions required for parametric tests.

## 3. Results

### 3.1. Legality Assessment of the Bowlers

**Figure 6:**
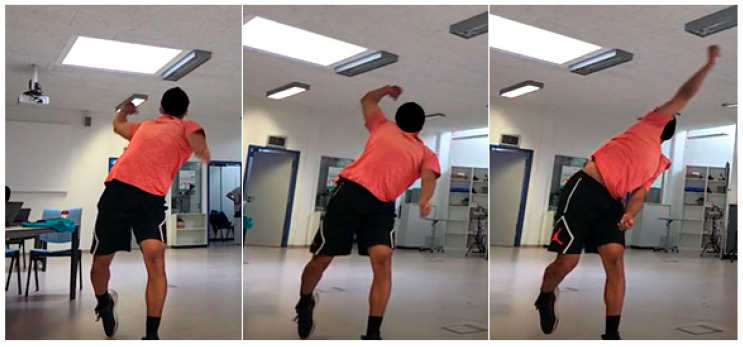
*Pre-intervention bowling action of Participant A.* The sequence illustrates the qualitative characteristics of the bowler’s action prior to the intervention, corresponding to the parameters outlined in Table 1. This pre-intervention technique displays the key features identified by the professional coach as contributing to an illegal bowling action.

**Figure 7:**
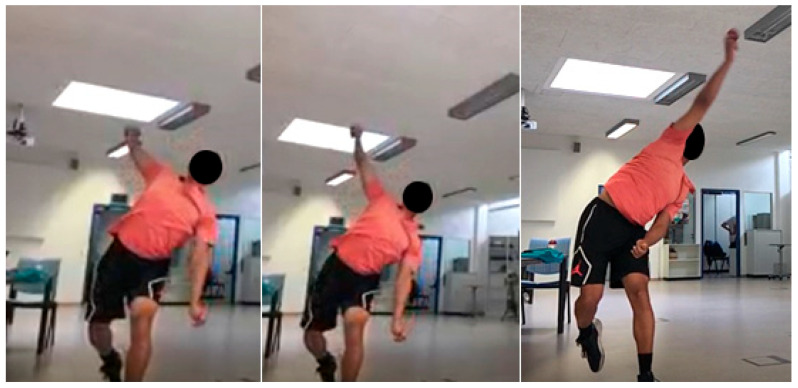
*Post-intervention bowling action of Participant A.* The sequence depicts the bowler’s modified action following the intervention, demonstrating the key qualitative changes described in Table 2. The contrasting technique highlights the improvements in elbow control and overall legality. Minor differences in camera orientation arose from the need to capture higher-resolution imagery during post-intervention recording, preventing identical oblique viewing angles.

Both bowlers exhibited substantial reductions in weighted illegality scores following the intervention. Participant A showed a 73.7% improvement (from 76 to 20; Table 1), while Participant B improved by 79.2% (from 53 to 11; Table 2). The greatest improvements were observed in the elimination of excessive elbow extension, reduction of visible elbow flexion during maximal shoulder external rotation, and improvements in trunk control and stride alignment. Notably, Participant B registered a lower pre-intervention score (Pre-B) than Participant A (Pre-A), indicating a less suspect action prior to the intervention. Although the current analysis is limited by single-trial ratings, the strong directional changes suggest the effectiveness of the technical intervention. The action characteristics of Bowler A are shown pre- and post-intervention (Figure 6 and Figure 7). The corresponding video footage for Participant B did not meet the resolution and image quality standards required for publication.

### 3.2. Integral of the Precession

**Figure 8:**
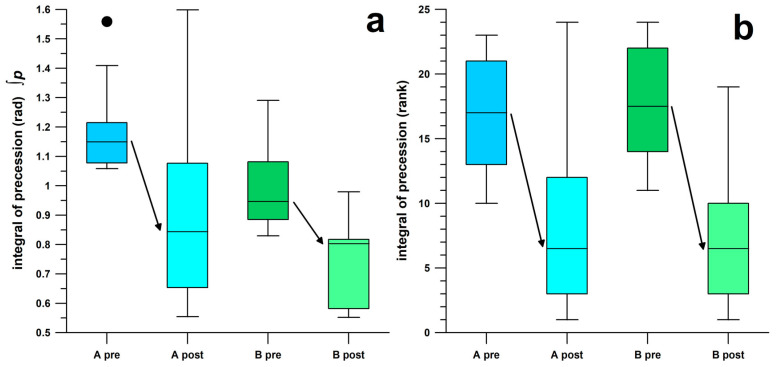
Box and Whisker plots of the integral of precession ∫*p*, (**a**) in radians and (**b**) as ranks; A and B: participant A and B; pre & post: data before & after the intervention; ●: outlier.

For both participants, the precession integral (∫*p*) decreased significantly (27% and 15%) over the time window *W* (Figure 2 and Figure 8; Table 3), corresponding to a reduction in observable elbow extension. Participant B showed better ∫*p*-data than participant A, which reflected the pre-intervention observation that participant B bowled with less elbow extension than participant A. The width of the time window*, W*, did not change after the intervention (*p* > 0.28; Table 3), a key finding indicating that ∫*p* was not influenced by *W*, providing evidence that ∫*p* depended solely on the amount of elbow angle. The time window *W* of participant B was significantly longer by 0.01 s (*p* < 0.001; r = 0.97) before and after the intervention than that of participant A, showing that participant B was able to impart torque on the ball for a longer period of time. In Figure 8a, the ∫*p*-range (overall and interquartile) of participant A is substantially larger after the intervention than before the intervention and the maximum ∫*p*-data did not decrease after the intervention (in contrast to participant B). This result may be due to participant A’s movements being less consistent with a flatter learning curve than those of participant B.

### 3.3. Physical and Skill Performance Parameters

Any change in physical or skill performance parameters is regarded as a side effect of the intervention.

For participant A, the spin rate *ω* decreases significantly after the intervention (Table 4) by 10.5%. This effect is reflected in the data of participant A, namely the decreasing *T_s_*__max_ and *α*_max_ (by 10%) while *p*_max_ and *p_n__*_max_ improve significantly (by approximately 25%; Table 4). Furthermore, the ratio of *T_s_*__max_ to *T_p_*__max_ decreases significantly by 16%. At first glance, this trend seems to be disadvantageous since less spin torque *T_s_* and more *T_p_* prevent the generation of angular kinetic energy, but paradoxically, *T_s_*__max_/*T_p_*__max_ is associated with effective conversion of torque to spin rate [49].

For participant B, the performance parameters are not affected by the intervention. However, three skill parameters, *p*_max_, *p_n__*_max_ and *α*/*ω* improve significantly (by 53%, 44%, and 7%, respectively). In addition, *ω/T_R_*__max_ improves by 8%, a factor that is related to the conversion of torque to spin rate.

### 3.4. Position of the Centre of Pressure

**Figure 9:**
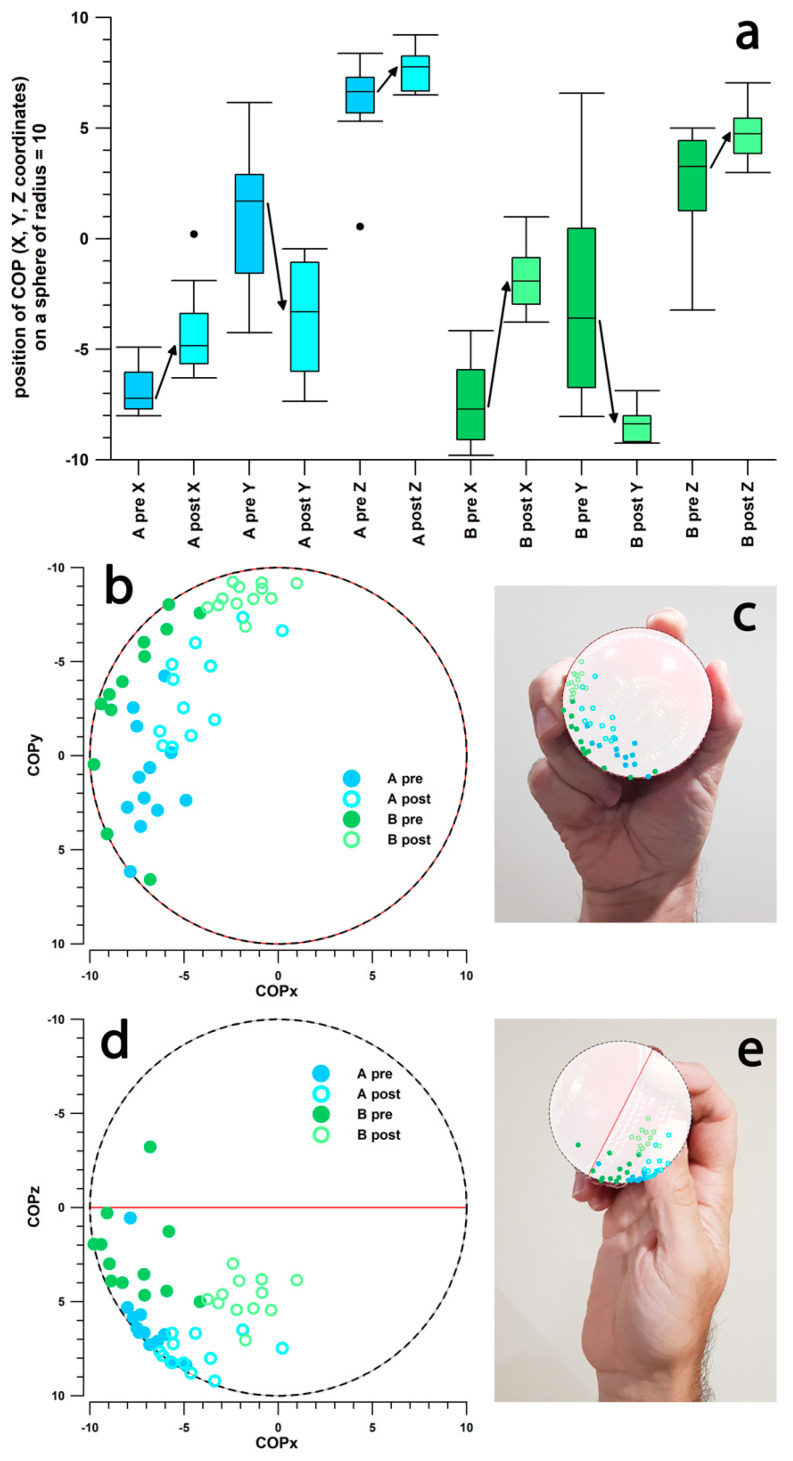
Position of the COP on the surface of the ball (radius = 10); (**a**) Box and Whisker plots of x-, y-, and z-coordinates of the COP (arrows indicate a significant change); A and B: participant A and B; pre & post: data before & after the intervention; ●: outlier; (**b**–**e**) x-, y-, and z-coordinates of the COP, projected on the xy- (**b**,**c**) and xz-planes (**d**,**e**); red circle (**b**,**c**) and red line (**d**,**e**): position of the seam.

Any change in the COP position is also considered a side effect of the intervention. In fact, changes in the three COP coordinates (x,y,z) over time were significantly different after the intervention (Table 5, Figure 9). After the intervention, the COP moved clockwise along the seam from the batter’s perspective (Figure 9b,c), i.e., in the direction opposite to the direction the ball spins as it left the hand (righthanded finger-spinner; Figure 9c). In the z-direction (Figure 9d,e), the COP moved closer to the north pole of the ball (negative z-direction, pointing toward the palm in righthanded finger-spinners; Figure 9e). The overall angular movement of the COP vector (COP_x,y,z_ in Table 5) during the intervention was 33.3° in participant A and 47.5° in participant B.

### 3.5. Type of Delivery

**Figure 10:**
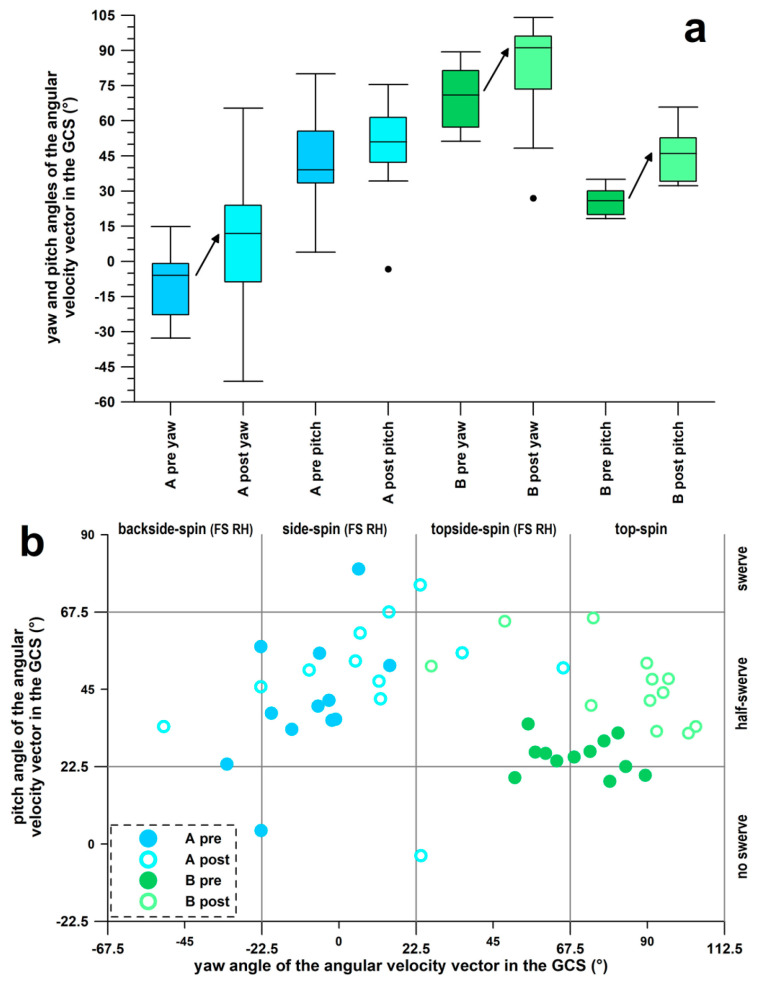
Type of delivery identified from the yaw and pitch angles of the angular velocity vector in the GCS (global coordinate system) immediately after release; (**a**) Box and Whisker plots of yaw and pitch angles (arrows indicate a significant change); A and B: participant A and B; pre & post: data before & after the intervention; ●: outlier; (**b**) pitch vs. yaw angle; FS RH = finger-spinner, righthander.

Both participants belonged to the generic category of off-spin bowlers. Participants A and B’s stock balls were classified as side-spin and top-spin types, respectively (Table 6). The median of the ω-yaw angle of participant A before and after the intervention was within the side-spin sector (yaw: 0° ± 22.5°; Figure 10), but after the intervention, the median yaw angle shifted significantly clockwise (to the right) by 16°. The median of the ω-pitch angle of participant A was within the half-swerve sector (45° ± 22.5°) and tilted insignificantly by +12° after the intervention.

The median of the ω-yaw angle of participant B was marginally (by 3.4°) before and exactly (at 91.1°) within the top-spin sector (yaw: 90° ± 22.5°; Figure 10) after the intervention, however, the median yaw angle shifted significantly by 20° counterclockwise (to the left and back; Table 6, Figure 10). The median of the ω-pitch angle of participant B was slightly (around 3.4°) before and exactly (at 46.0°) within the half-swerve sector (45° ± 22.5°; Figure 10) after the intervention. However, the median yaw angle correspondingly became significantly steeper by 20.2° (Table 6, Figure 10).

## 4. Discussion

This study demonstrated that the technical interventions designed to remediate illegal bowling actions were effective, producing measurable changes in primary performance parameters while also affecting secondary parameters not directly targeted. Consistent with the study’s aims, the smart cricket ball was used to evaluate bowling performance from multiple perspectives, capturing physical and skill parameters, centre of pressure position, spin delivery type in relation to illegal bowling actions. The correction of the illegal actions was visually confirmed by a professional cricket coach (REDF) using slow-motion video from multiple angles, ensuring that observed changes in technique aligned with the intervention goals.

This visual confirmation of a reduction of elbow extension in the bowlers corresponded with the changes observed in the novel parameter of precession, particularly the integral of precession (∫*p*) measured via the smart ball. Both participants experienced a significant decrease in (∫*p*) during the designated time window *W* (Figure 2 and Figure 8; Table 3), while the width of *W* remained largely unchanged, indicating that the timing of torque transfer to the ball was not affected at all. These results align with the study’s aim of comparing pre- and post-intervention performance parameters, not only for the targeted outcome but also for secondary effects on non-targeted parameters.

Further confirmation of changes in the bowlers’ physical characteristics is evident in the box-and-whisker plots of Figure 8, showing the rank distributions before and after intervention. These ranks were determined through the Mann–Whitney U test, comparing each group’s medians and interquartile ranges. For the integral of precession, the interquartile ranges of the pre-intervention ‘suspect’ actions and post-intervention legal actions did not overlap, producing large effect sizes (Table 3). Consequently, the effect sizes shown in Table 3 for these data are quite large. Hence, the distinct lack of overlap in the interquartile ranges between the pre-intervention ‘suspect’ bowling actions and post-intervention legal bowling actions regarding the integral of precession supports the effectiveness of the intervention through changes in precession (∫*p*, significant reduction of 15–27%), suggesting a tangible refinement in the bowlers’ technique.

Unlike traditional methods that rely on laboratory-based measurements with markers, which can impede performance, the smart ball can measure ∫*p* in the field of play, accurately capturing the nuances of the bowling action in its natural environment. If these results generalise to the wider bowling population, excessive elbow extension would increase the precession torque applied to the ball, causing a greater deviation in the spin vector. This suggests that the effectiveness of a coaching intervention aimed at reducing elbow extension can be established by a corresponding decrease in ∫*p*. Consequently, this metric could emerge as a valuable tool for both analysing and coaching cricket bowling, supporting the enforcement of fair play.

The novel coaching intervention that reduced ∫*p* in this study incorporated bowling techniques that challenge the traditional coaching models for finger-spinners, which typically advocate standing tall, emphasise a very short delivery stride, and promote an early aggressive drive of the rear knee forward, presumable to accelerate the bowling arm and assist in the generation of spin rate. However, this conventional approach contradicts fundamental biomechanical principles, particularly the kinetic link principle, which relies on ground-reaction braking impulses through the front foot [44]. On the contrary, the coaching intervention in this study trained the bowlers to take a longer stride, landing the front foot ahead of the centre of mass, enabling a substantial horizontal braking impulse. This impulse can be utilised to initiate a proximal-to-distal sequential transfer of energy from the pelvis to the bowling arm. The alternative is to rely more proportionately on power generation from the smaller distal joints, such as the elbow and wrist, to generate power [52], a less efficient option which may lead to a higher degree of elbow extension. Optimising whole-body mechanics is critical, as dynamic coupling in multi-segmental systems amplifies the efficiency of force transmission [34]. However, unlike traditional interventions that often overlook elbow mechanics [53], our approach emphasised whole-body dynamics while also specifically training new neuromuscular patterns to regulate elbow motion, potentially influenced by shoulder physiology and motor control [54].

Although not as extensively studied or well understood as passive insufficiency in the hip, preliminary research suggests that passive insufficiency also occurs in the shoulder [55]. This characteristic informed the design of an intervention targeting key biomechanical factors contributing to shoulder passive insufficiency during the bowling action: excessive shoulder external rotation, forearm supination, and wrist extension (or flexion, dependent on technique) during “arm-loading” behind the back. When the bowling arm extends horizontally behind the bowler’s back, some spin bowlers aiming to generate high spin rates can exhibit early and pronounced shoulder external rotation coupled with excessive forearm supination. This combination mimics the beginning of the arm-cocking (or arm-loading) phase in throwing, pre-loading and placing the shoulder internal rotators under eccentric pre-stretch [56]. To mitigate this strain in suspect spin bowlers, the elbow flexes as the arm moves past the horizontal position behind the back. This compensatory elbow flexion may result from passive insufficiency of the long head of the triceps brachii, which originates from the scapula. In some bowlers, an alternative technique involving an extended wrist can exacerbate this effect, as the wrist extensors contribute to further limiting the ability of the forearm and elbow to maintain extension, thereby increasing elbow flexion as the bowling arm approaches the horizontal position behind the back. Unfortunately, this premature flexion can trigger rapid forearm pronation and subsequent elbow extension during arm acceleration, substantially raising the risk of producing an illegal delivery in bowling.

It is important to emphasise that passive insufficiency at the shoulder indicates that interventions to correct an illegal bowling action should begin by limiting excessive shoulder external rotation and constraining elbow flexion (<15–20°). Bowlers should be instructed to maintain the shoulder in an anatomically neutral external rotation and to keep the elbow relatively extended as the upper arm approaches the horizontal position. This strategy provides a mechanically safe starting posture. However, other procedures must be followed to reduce the amount of elbow extension during the subsequent arm-acceleration phase.

These further procedures should consider the natural coupling between elbow extension and segment-dependent interactions, which operate in typical throwing-type motions, such as passive velocity-dependent torques and other interactive torque components [34,35,57]. To inhibit this natural coupling, our intervention emphasised Type 1 spin mechanics [36], instructing bowlers to generate off-spin through a coordinated pattern of forearm supination, wrist flexion, and finger flexion with inward twisting. Because wrist flexion occurs largely orthogonal to the ulnar-deviation plane, the resulting synergy effectively avoids the pronation-driven throwing pattern and thereby reduces both the mechanical and neuromuscular drive toward excessive elbow extension. After the bowler achieves a consistently legal action using this Type 1 pattern, more advanced Type 2 mechanics, which incorporate greater trunk and shoulder contributions, can be introduced if desired.

Biomechanical evidence supports this rationale. Electromyographic analysis shows that during wrist flexion, flexor carpi radialis (FCR) activity increases with forearm supination, whereas flexor carpi ulnaris (FCU) activity decreases with pronation, creating a coordination pattern favouring ulnar-directed flexion [58]. Conversely, during wrist extension, extensor carpi radialis brevis (ECRB) activity rises with forearm pronation, promoting ulnar-directed extension characteristic of the throwing motion [58]. Complementary torque measurements confirm that maximal wrist-flexion torque occurs in a supinated forearm, whereas wrist-extension torque peaks in pronation [47]. It has also been shown that forearm muscle activity can significantly alter the activity of the biceps and triceps brachii, affecting the local control of the elbow joint [59]. Nevertheless, this coaching intervention must clearly specify that the bowler maintains a relatively extended elbow with minimal shoulder external rotation, and delays forearm supination and wrist flexion until after the upper arm has passed the horizontal (Figure 1). Only if this condition is satisfied can rapid forearm supination and controlled wrist flexion potentially induce a coordinated movement synergy that potentially acts to reduce elbow extension [45].

Furthermore, a key feature of the intervention was the deliberate coordination of finger flexion with wrist flexion to generate spin torque to constrain elbow extension. In a controlled EMG study, Beringer et al. (2020) [48] showed that activation of the extrinsic finger flexors increased significantly when the wrist was held in flexion compared with extension, even when finger movement was minimised. This finding suggests that the intention to increase finger flexor recruitment will induce more flexed wrist posture, which in turn will synergistically act to inhibit or reduce elbow extension, creating a mechanical and neural synergy that links the three actions. The multi-faceted action of these intervention processes may explain the rapid transformative effect on the elbow mechanics of these two spin bowlers (Table 3, Table 4, Table 5 and Table 6), legalising their bowling actions within a 30-min intervention (Table 1 and Table 2) without a significant decrement in performance (Table 4).

When implementing a coaching intervention to correct an illegal bowling action, it is critical to evaluate both primary and secondary biomechanical outcomes, ensuring that any unintended effects align with the goals of enhancing performance or reducing injury risk. Neglecting secondary outcomes could unintentionally limit the intervention’s effectiveness or create new injury risks. In this study, we observed changes in several performance parameters in response to the intervention for correcting illegal bowling actions. These included peak precession and peak normalised precession for both participants, all three centre of pressure (COP) coordinates, and the yaw angle of the angular velocity vector, which characterises the type of delivery in the horizontal plane of the Global Coordinate System (GCS). Additionally, some performance parameters exhibited changes in only one participant, such as the pitch angle of the angular velocity vector for Participant B: *ω*_max_, *T_s_*__max_, *α*_max_ and *T_s_*__max_/*T_p_*__max_ for participant A; and *α*_max_/*ω*_max_ and *ω*/*T_R_* for participant B.

Specifically, for Participant A, the coaching intervention to reduce elbow extension was successful, but at the cost of a 10.5% decrease in spin rate (ω). This is expected in the short term due to the learning effect associated with modifying a long-term movement pattern, as it is common for performance to temporarily decline when an individual adapts to a newly introduced technique [60]. However, the reduction in spin rate was surprisingly small, especially considering the substantial adjustments made to the bowling action to transform an illegal technique into a legal one. In addition to the decrease in spin rate, both the maximum spin torque (*T_s_*__max_) and the maximum angular acceleration (*α*_max_) also showed reductions, indicating that less spin torque was being applied to the ball and that the ball’s angular acceleration had diminished after the intervention. Despite the anticipated short-term reductions, the relatively modest change in spin rate suggests that the bowler was able to maintain a level of performance even after significant modifications to the action.

In addition, there was a notable improvement in the efficiency of the bowling action, as indicated by the significant reduction in both maximum precession velocity (*p*_max_) and normalised precession (*p_n_*) by 25–53%. Precession refers to the change in the direction of the spin vector, and the improvement in these parameters suggests that the spin vector was changing less throughout the bowling action. This reduction in precession is a promising indicator of the intervention’s effectiveness in improving the stability and control of the bowling action. Additionally, this improvement in precession, alongside the small reduction in spin rate, suggests that with more time and further adaptation, the spin rate may increase as the bowler becomes more proficient with the new technique. This overall improvement in efficiency could also indicate the potential for long-term gains in performance.

In contrast, for participant B, the coaching intervention had little effect on most performance parameters. This suggests that the overall performance of participant B’s bowling action remained stable after the intervention. However, there were still significant improvements in *p*_max_, *p_n_*, and *α*/*ω* (by 53%, 44%, and 7%, respectively). In addition to these improvements, the ratio of spin rate to the maximum time of release (*ω*/*T_R_*__max_) also improved significantly by 8%. This indicates an improvement in the conversion of torque to spin rate. In other words, the intervention helped participant B to convert the torque applied during the bowling action more efficiently into spin on the ball. This could potentially lead to a more consistent and effective bowling action in the long term.

Another secondary outcome of the coaching intervention was related to the type of spin delivery. Bowlers A and B were not pure side- and top-spinners, respectively (Table 6, Figure 10), but had their spin axis tilted into the half-swerve zone (Figure 10) with respect to the plane of the seam. After the intervention, Bowler B increased the ω-pitch angle, resulting in an enhanced lateral swerve component of the ball due to the Magnus effect. This creates an extra dimension to the ball’s trajectory, adding a lateral variable in flight. According to the motor control theory of human perception, any additional degrees of freedom in the ball’s flight path would increase the complexity of the batter’s task [61].

When players adopt a new or adjusted bowling technique with technical help, they often experience a temporary drop in some physical abilities, even though their skill level improves. This is a common occurrence in sports training, where the body needs time to adjust to a new way of moving. A study by Fuss et al. [27] illustrates this phenomenon. They aimed to improve the efficiency of a finger-spinner who specialised in top-side-spin deliveries. Before the training, the bowler’s efficiency *η* averaged at 39%. The training involved learning a new bowling technique. After three hours of corrective training this led to a further decrease in five physical performance measures: *ω*, *T_R_*, *T_s_*, *α*, *P*. However, despite this initial setback, the training successfully improved the bowler’s efficiency *η* to 59%. Additionally, three other skill measures also showed significant improvement: *p_n_*, *T_p_*__max_ and *α*_max_/*ω*_max_. In summary, while adopting a new or adjusted bowling technique may initially reduce some physical abilities, it can ultimately lead to significant improvements in skill over time.

The novelty of this study lies in its new and holistic approach to intervention measures. This holistic approach is defined by the fact that not only is the outcome of the target parameter measured and the data used to assess the success or failure of the intervention, but other, non-target-oriented parameters are also examined to determine the extent to which they are influenced by the intervention. In this context, the target parameter is may be completely decoupled from the non-target parameters, since changes in the latter do not necessarily depend on the success of the former. Previous research by the authors [2] indicated that the psychological intention alone to increase spin rate was sufficient to modify bowling technique by improving normalised precession, even if the spin rate did not actually increase. This principle creates the possibility of verifying whether an intervention actually took place, even if it is unsuccessful. As long as any other parameter not related to the target shows a significant change after the intervention, the mechanical response to the intervention is considered to be confirmed. In this particular study, it is irrelevant whether or not the intervention improved the illegal throwing technique (Figure 6 and Figure 7), as a variety of parameters changed significantly due to the intervention, all of which were measured with a single measuring device: the smart cricket ball. Preferably, all parameters (target, non-target) should be measured with the same measurement device, e.g., a motion capture system, or, in the present case, a smart cricket ball. Although a direct measurement of the elbow extension angle to detect a rule violation above the 15° limit [62] was not the aim of this study, the smart cricket ball offers the possibility of qualitatively assessing rule violations in bowling using another parameter developed in a previous study [28]. The results of this study are briefly explained in the Introduction section. Any movement beyond the standard bowling motion imparts a torque on the cricket ball, which is detected by the smart cricket ball. If the torque vector **T_R_** is not parallel to the **ω**-vector, the **T_R_**-component, namely **T_P_**, causes a movement of the **ω**-vector across the ball’s surface, a phenomenon known as precession (*p*). This principle applies to rule violations in bowling, as extension movements of the forearm cause a precession spike [28]. The magnitude of *p*, more precisely the time integral ∫*p* of *p*, corresponding to the movement of **ω**-vector in radians, calculated from the smart ball data, is thus a representative measure of the torque generated by the elbow extension, which qualitatively corresponds to the degree of illegal bowling. The term “qualitative” is to be understood analogously to the magnitude of an EMG signal, which qualitatively corresponds to the force of a muscle [63], i.e., the higher the amplitude, the greater the force. The non-linearity of this relationship is not considered here, unless a calibration curve has been established [64], as shown in Figure 7 of De Luca’s publication [63]. These calibration curves differ depending on the muscle [63]. The same principle applies to the time integral ∫*p* of *p*: a calibration curve of elbow extension angle vs ∫*p* is required, with the extension angle being measured, for example, using a motion capture system. This has the disadvantage that small extension angles are only available after the intervention. Nevertheless, the principle “the larger the extension angle, the greater ∫*p*” is applicable and has been validated against three-dimensional motion analysis systems capable of detecting elbow extension beyond the ICC’s 15° threshold [28]. As an expected result, the precession integral ∫*p* responded to the intervention and showed significant improvements (Table 3, Figure 8).

This study uniquely integrates an ecologically valid measurement system with a structured coaching intervention that constrains elbow extension in concert with movement synergies to identify and correct suspect bowling actions in a practical, field-based setting. By combining quantitative diagnostics with targeted technique modification, it extends the application of the smart ball beyond performance profiling to the domain of action legality and corrective coaching, which is a substantial advancement over conventional field-based approaches available to coaches. To ease the difficulty for coaches, the following stepwise framework is proposed:

1. Initial Visual Assessment: Conduct video-based analysis from multiple angles, focusing on the 14 key biomechanical parameters relevant to legality.
2. Baseline Smart Ball Testing: Require the bowler to deliver one over (six balls) to establish pre-intervention performance and legality data.
3. Intervention and Feedback: Run a technical remediation session (30 min minimum), providing explicit feedback to reduce elbow extension and enhance coordinated forearm supination and wrist flexion.
4. Post-Intervention Assessment: Repeat the smart-ball deliveries to evaluate changes in precession and other relevant metrics.
5. Ongoing Monitoring: Periodically reassess to ensure retention of the corrected action and long-term legality.

Such a framework offers coaches a clear, practical protocol for objectively identifying, correcting, and verifying suspect spin bowling actions, even in small cohorts, without relying solely on visual observation.

The limitations of this study are twofold.

(1) The main limitation of this study is sample size (only two participants), which restricts the statistical power of the analyses and limits the generalisability of the findings. As such, the results should be considered preliminary and interpreted with caution. Nevertheless, the clear directional changes in elbow extension and smart-ball parameters provide encouraging evidence for the feasibility of the intervention. As focus was on the side effects of the intervention, irrespective of the outcome of the intervention itself, we compared the data of each parameter before and after the intervention with the MWU-test to detect any significant differences for each bowler individually. The focus of this study was not to compare different bowlers based on side effects which we did not know in the first place. If the number of side-effects were scarce and inconsistent between bowlers, a larger sample were justified to present the case of side-effects. Larger trials are needed to confirm these effects and determine whether the observed improvements can be reproduced in a broader population of spin bowlers. Notably, changes were detected not only in the primary target variable (∫*p*), but also in secondary measures, including the position of the centre of pressure (COP), the yaw angle of the angular velocity vector in the global coordinate system (GCS), and two skill-related parameters: precession and normalised precession. In addition, even with small subject numbers, the smart ball offers a means of validating a coach’s claims that a prescribed technical intervention has indeed led to tangible changes, rather than relying solely on self-report.
(2) Furthermore, since the intervention trials were conducted outdoors, direct measurement of elbow flexion angles was not feasible. Instead, changes in elbow motion were inferred from the smart cricket ball data, as rapid elbow extension during delivery influences the magnitude of the precession integral (∫*p*). To complement these indirect measurements, each delivery was recorded on a smartphone during and after the intervention. The recordings were reviewed immediately after each delivery by an experienced bowler and cricket coach (REDF). Bowling legality was subsequently evaluated using a newly developed qualitative assessment tool comprising 14 parameters (Table 1 and Table 2).

These types of intervention studies and coaching procedures, as described in this study, could not be performed with a simple smart cricket ball such as the Kookaburra ball, “*not available for sale yet*” [64], as it does not measure the angular velocity of the ball using high-speed gyroscopes but estimates the spin rate from the frequency of magnetometer signals during flight [65]. It therefore only provides a single spin rate value at ball release. Spin rate alone is not sufficient for performance analysis, as shown in Table 4. Calculating these new performance parameters requires a continuous measurement of the angular velocity at a high sampling frequency [27], and this is equally necessary for calculating the COP [51] (Table 5, Figure 9) and the type of delivery [29] (Table 6, Figure 10).

The future projections of our work with the advanced smart cricket ball are to set a new standard for profiling, performance assessment, and coaching in spin bowling, which is considered a lost art [27]. The challenges we want to address in this context are to take spin bowling to the next level by teaching spin bowlers the variety of deliveries using the smart cricket ball, allowing bowlers to analyse their standard balls and increase their repertoire of deliveries to not only improve performance, but to more deeply appreciate the complex art of spin bowling.

## 5. Conclusions

This study demonstrates that the advanced smart cricket ball is a powerful tool for assessing the effectiveness of coaching intervention to correct illegal actions, precisely quantifying both primary and secondary changes in performance. Its portability and ability to function outside laboratory settings provide a practical, real-world solution for evaluating and correcting illegal bowling actions in cricket, enabling interventions to be guided by scientific data. Beyond remediation, the smart ball offers significant potential for enhancing spin bowling performance by capturing detailed ball dynamics metrics, as well as the interactions between these elements during delivery. By revealing how technical adjustments affect the ball, including secondary effects, the smart ball assists coaches in fine-tuning their training protocols to improve performance. Consequently, we recommend that coaching interventions not only target the primary performance parameters in spin bowlers but also systematically monitor secondary effects, using consistent pre- and post-intervention measurements, to maximise both skill development and adherence to the rules of the game.

## Figures and Tables

**Table 1:** Pre-Intervention Weighted Scores by Assessment Item; Wt = Item weighting; Ratings: 0 = Not observed to 4 = Clearly observed; Pre-A = Rating pre-intervention for Participant A; Pre-B = Rating pre-intervention for Participant B; Pre-A Wt = Pre-A × Wt; Pre-B Wt = Pre B × Wt. Maximum possible score = 76; ER = external rotation.

Assessment Item	Wt	Pre A	Pre A Wt	Pre B	Pre B Wt
Excessive elbow extension during arm-acceleration	3	4	12	3	9
Jerky or whipping motion at release	1	4	4	3	3
Excessive shoulder external rotation	2	4	8	3	6
Scapular-thoracic motion ± lumbar extension	1	4	4	3	3
Visible elbow flexion at maximal shoulder ER	3	4	12	4	12
Early shoulder abduction	1	4	4	4	4
Bowling arm above vertical at release	1	4	4	2	2
Release point significantly ahead of front foot	1	4	4	1	1
Minimal trunk flexion at release	1	4	4	1	1
Minimal “V-angle” (trunk–front thigh)	1	4	4	1	1
Front-on alignment at back foot contact	1	4	4	4	4
Excessive lateral bending ± shoulder rotation	1	4	4	0	0
Open stride angle ± open foot position	1	4	4	4	4
Stride length < 1.2 × shoulder width (short)	1	4	4	3	3
**Total Score (sum)**			**76**		**53**

**Table 2:** Post-Intervention Weighted Scores by Assessment Item; Wt = Item weighting; Ratings: 0 = Not observed to 4 = Clearly observed; Post-A = Rating post-intervention for Participant A; Pre-B = Rating post-intervention for Participant B; Post-A Wt = Post-A × Wt; Pre-B Wt = Post-B × Wt. Maximum possible score = 76; ER = external rotation.

Assessment Item	Wt	Post-A	Post-A Wt	Post-B	Post-B Wt
Excessive elbow extension during arm-acceleration	3	1	3	0	0
Jerky or whipping motion at release	1	1	1	0	0
Excessive shoulder external rotation	2	1	2	0	0
Scapular-thoracic motion ± lumbar extension	1	2	2	0	0
Visible elbow flexion at maximal shoulder ER	3	1	3	0	0
Early shoulder abduction	1	1	1	1	1
Bowling arm above vertical at release	1	1	1	0	0
Release point significantly ahead of front foot	1	1	1	0	0
Minimal trunk flexion at release	1	1	1	0	0
Minimal “V-angle” (trunk–front thigh)	1	0	0	1	1
Front-on alignment at back foot contact	1	0	0	2	2
Excessive lateral bending ± shoulder rotation	1	2	2	1	1
Open stride angle ± open foot position	1	3	3	4	4
Stride length < 1.2 × shoulder width	1	0	0	2	2
**Total Score (sum)**			**20**		**11**

**Table 3:** Statistics of integral of precession (∫*p*); *W*, width of time window at ¼ of maximum *T_R_* (Figure 2); med-pre, med-post, medians before and after intervention; r, effect size; ⊥, no change (if *p* > 0.05); ↑, improvement.

	∫*p* (rad)	Δ*t* = *W* (s)	∫*p* (rad)	Δ*t* = *W* (s)
	Participant A		Participant B	
med-pre	1.150	0.061	0.946	0.050
med-post	0.844	0.059	0.803	0.050
*p*-value	**0.003**	0.285	**<0.001**	0.582
r	0.72	0.26	0.88	0.14
effect	large	medium	large	small
trend	**↑**	⊥	**↑**	⊥

**Table 4:** Statistics of performance parameters; medians before (med-pre) and after intervention (med-post); r, effect size; *ω*, angular velocity; *p*, precession; *p_n_*, normalised precession; *T_R_*, resultant torque; *T_s_*, spin torque; *T_p_*, spin torque; *α*, angular acceleration; *P*, power; *η*, efficiency; max, maximum; ⊥, no change (if *p* > 0.05); ↑, improvement; ↓, deterioration.

	*ω*_max_ (rad/s)	*p*_max_ (rad/s)	*p_n__*_max_ (°)	*T_R_*__max_ (Nm)	*T_s_*__max_ (Nm)	*T_p_*__max_ (Nm)	*α*_max_ (rad/s^2^)	*P*_max_ (W)	*η* (%)	*α*/*ω* (s^−1^)	*ω/T_R_*__max_	*T_s_*__max_ */T_p_*__max_
*participant A*												
med-pre	127.7	47.9	100.3	0.223	0.204	0.092	2629	15.6	65.4	21.6	87.9	2.15
med-post	114.3	35.6	75.7	0.198	0.184	0.098	2365	12.7	65.5	20.6	88.2	1.80
*p*-value	**0.040**	**<0.001**	**0.007**	0.054	**0.047**	0.285	**0.047**	0.060	0.795	0.069	0.435	**0.004**
r	0.50	0.86	0.65	0.47	0.49	0.26	0.49	0.46	0.07	0.44	0.19	0.69
effect	**large**	**large**	**large**	**large**	**large**	medium	**large**	**large**	very small	**large**	small	**large**
trend	↓	**↑**	**↑**	⊥	↓	⊥	↓	⊥	⊥	⊥	⊥	↓(**↑**)
*participant B*												
med-pre	191.2	48.8	131.3	0.378	0.374	0.080	4813	39.2	75.9	25.1	80.4	4.75
med-post	195.0	23.1	73.5	0.362	0.359	0.102	4617	38.4	77.5	23.4	86.6	3.65
*p*-val	0.542	**<0.001**	**<0.001**	0.112	0.112	0.215	0.112	0.624	0.099	**<0.001**	**<0.001**	0.215
r	0.15	1	1	0.39	0.39	0.31	0.39	0.13	0.40	0.94	0.96	0.31
effect	small	**large**	**large**	**large**	**large**	medium	**large**	small	**large**	**large**	**large**	medium
trend	⊥	**↑**	**↑**	⊥	⊥	⊥	⊥	⊥	⊥	**↑**	**↑**	⊥

**Table 5:** Statistics of COP; medians before (med-pre) and after intervention (med-post); *p*-val, *p*-value; r, effect size; COPx, COPy, COPz, position of the centre of pressure in x, y, z directions on the surface of a sphere of a radius *r* of 10 (arbitrary units).

	COPx (*r* = 10)	COPy (*r* = 10)	COPz (*r* = 10)	COPx (*r* = 10)	COPy (*r* = 10)	COPz (*r* = 10)
	Participant A			Participant B		
med-pre	−7.22	1.70	6.64	−7.71	−3.60	3.27
med-post	−4.84	−3.30	7.76	−1.91	−8.37	4.74
*p*-val	**<0.001**	**0.002**	**0.016**	**<0.001**	**<0.001**	**0.010**
r	0.86	0.76	0.58	1	0.94	0.63
effect	large	large	large	large	large	large

**Table 6:** Statistics of the type of delivery (yaw and pitch angles of the angular velocity vector in the GCS); medians before (med-pre) and after intervention (med-post); *p*-val, *p*-value; r, effect size.

	Yaw Angle (°)	Pitch Angle (°)	Yaw Angle (°)	Pitch Angle (°)
	Participant A		Participant B	
med-pre	−5.93	39.09	70.94	25.88
med-post	11.91	50.97	91.08	46.03
*p*-val	**0.035**	0.194	**0.030**	**<0.001**
r	0.51	0.32	0.53	0.94
effect	**large**	medium	**large**	**large**
type of delivery	finger-spin side-spin	half-swerve	finger-spin top-spin	half-swerve

## Data Availability

The raw data supporting the conclusions of this article will be made available by the authors to qualified researchers, if they have obtained Ethics Approval for secondary use of existing data through a Consent Waiver.

## References

[1] Oslear D. (2000). Wisden’s The Laws of Cricket.

[2] Ferdinands R.E., Kersting U.G. (2007). An evaluation of biomechanical measures of bowling action legality in cricket. Sports Biomech..

[3] Middleton K.J., Alderson J.A., Elliott B.C., Mills P.M. (2015). The influence of elbow joint kinematics on wrist speed in cricket fast bowling. J. Sports Sci..

[4] Marshall R.N., Ferdinands R. (2003). The effect of a flexed elbow on bowling speed in cricket. Sports Biomech..

[5] Marshall R.N., Elliott B.C. (2000). Long-axis rotation: The missing link in proximal-to-distal segmental sequencing. J. Sports Sci..

[6] International Cricket Council (ICC) (2018). ICC Regulations for the Review of Bowlers Reported with Suspected Illegal Bowling Action.

[7] International Cricket Council (ICC) (2024). Illegal Bowling Actions.

[8] Portus M.R., Rosemond C.D., Rath D.A. (2006). Cricket: Fast bowling arm actions and the illegal delivery law in men’s high performance cricket matches. Sports Biomech..

[9] Felton P.J., King M.A. (2016). The effect of elbow hyperextension on ball speed in cricket fast bowling. J. Sports Sci..

[10] Aginsky K.D., Noakes T.D. (2010). Why it is difficult to detect an illegally bowled cricket delivery with either the naked eye or usual two-dimensional video analysis. Br. J. Sports Med..

[11] Lloyd D.G., Alderson J., Elliott B.C. (2000). An upper limb kinematic model for the examination of cricket bowling: A case study of Mutiah Muralitharan. J. Sports Sci..

[12] King M.A., Yeadon M.R. (2012). Quantifying elbow extension and elbow hyperextension in cricket bowling: A case study of Jenny Gunn. J. Sports Sci..

[13] Spratford W., Elliott B., Portus M., Brown N., Alderson J. (2018). Illegal bowling actions contribute to performance in cricket finger-spin bowlers. Scand. J. Med. Sci. Sports.

[14] Spratford W., Alderson J., Elliott B. Illegal bowling actions laws, do they really matter?. Proceedings of the 36th Conference of the International Society of Biomechanics in Sports.

[15] Turner J.A., Chaaban C.R., Padua D.A. (2024). Validation of OpenCap: A low-cost markerless motion capture system for lower-extremity kinematics during return-to-sport tasks. J. Biomech..

[16] Wixted A., Portus M., James D. (2010). Virtual & inertial sensors to detect illegal cricket bowling. Proc. Eng..

[17] Wixted A., James D., Busch A., Portus M. (2010). Wearable sensors for the monitoring of bowling action in cricket. J. Sci. Med. Sport.

[18] Wixted A., Spratford W., Davis M., Portus M., James D. Wearable sensors for on field near real time detection of illegal bowling actions. Proceedings of the Conference of Science, Medicine and Coaching in Cricket 2010.

[19] Wixted A., James D., Portus M. (2011). Inertial sensor orientation for cricket bowling monitoring. IEEE Sens..

[20] Wixted A., Portus M., Spratford W., James D. (2011). Detection of throwing in cricket using wearable sensors. Sports Technol..

[21] Qaisar S., Imtiaz S., Glazier P., Farooq F., Jamal A., Iqbal W., Lee S., Murgante B., Carlini S.M.M., Torre C.M., Nguyen H.-Q., Taniar D., Apduhan B.O., Gervasi O. (2013). A method for cricket bowling action classification analysis using a system of inertial sensors. Computational Science and Its Applications.

[22] Salman M., Qaisar S., Qamar A.M. (2017). Classification and legality analysis of bowling action in the game of cricket. Data Min. Knowl. Disc..

[23] Ahmed A., Asawal M., Khan M.J., Cheema H.M. A wearable wireless sensor for real time validation of bowling action in cricket. Proceedings of the 2015 IEEE 12th International Conference on Wearable and Implantable Body Sensor Networks (BSN).

[24] Goonetilleke R.S. (1999). Legality of bowling actions in cricket. Ergonomics.

[25] Fuss F.K., Smith R.M., Subic A. (2012). Determination of spin rate and axes with an instrumented cricket ball. Proc. Eng..

[26] Doljin B., Fuss F.K. (2015). Development of a smart cricket ball for advanced performance analysis of bowling. Proc. Techn..

[27] Fuss F.K., Doljin B., Ferdinands R.E.D. (2021). Mobile computing with a smart cricket ball: Discovery of novel performance parameters and their practical application to performance analysis, advanced profiling, talent identification and training interventions of spin bowlers. Sensors.

[28] Fuss F.K., Ferdinands R.E.D., Beach A., Doljin B. Detection of illegal bowling actions with a smart cricket ball. Proceedings of the 5th World Congress of Science & Medicine in Cricket.

[29] Fuss F.K., Doljin B., Ferdinands R.E.D. (2024). Identification of Spin Bowling Deliveries with a Smart Cricket Ball. Sensors.

[30] Miyazaki S., Yamako G., Totoribe K., Sekimoto T., Kadowaki Y., Tsuruta K., Chosa E. (2021). Shadow pitching deviates ball release position: Kinematic analysis in high school baseball pitchers. BMC Sports Sci. Med. Rehabil..

[31] Maker R., Taliep M.S. (2021). The effects of a four weeks combined resistance training programme on cricket bowling velocity. S. Afr. J. Sports Med..

[32] Myers J.B., Pasquale M.R., Laudner K.G., Sell T.C., Bradley J.P., Lephart S.M. (2005). On-the-Field Resistance-Tubing Exercises for Throwers: An Electromyographic Analysis. J. Athl. Train..

[33] Ramalho A., Petrica J. (2023). Knowledge in motion: A comprehensive review of evidence-based human kinetics. Int. J. Environ. Res. Public Health.

[34] Naito K., Takagi T., Kubota H., Maruyama T. (2017). Multi-body dynamic coupling mechanism for generating throwing arm velocity during baseball pitching. Hum. Mov. Sci..

[35] Hirashima M., Yamane K., Nakamura Y., Ohtsuki T. (2008). Kinetic chain of overarm throwing in terms of joint rotations revealed by induced acceleration analysis. J. Biomech..

[36] Beach A.J., Ferdinands R.E., Sinclair P.J. (2018). The relationship between segmental kinematics and ball spin in Type-2 cricket spin bowling. J. Sports Sci..

[37] Miyashita K., Urabe Y., Kobayashi H., Yokoe K., Koshida S., Kawamura M., Ida K. (2008). The role of shoulder maximum external rotation during throwing for elbow injury prevention in baseball players. J. Sports Sci. Med..

[38] Crotin R.L., Slowik J.S., Brewer G., Cain E.L., Fleisig G.S. (2022). Determinants of biomechanical efficiency in collegiate and professional baseball pitchers. Am. J. Sports Med..

[39] Konda S., Yanai T., Sakurai S. (2015). Configuration of the shoulder complex during the arm-cocking phase in baseball pitching. Am. J. Sports Med..

[40] Fritch J., Parekh A., Labbe A., Courseault J., Savoie F., Longo U.G., De Salvatore S., Candela V., Di Naro C., Casciaro C., Koh J., Zaffagnini S., Kuroda R., Longo U.G., Amirouche F. (2021). Biomechanics of the Throwing Shoulder. Orthopaedic Biomechanics in Sports Medicine.

[41] Singh H., Lee M., Solomito M.J., Merrill C., Nissen C. (2018). Lumbar Hyperextension in Baseball Pitching: A Potential Cause of Spondylolysis. J. Appl. Biomech..

[42] Oyama S., Yu B., Blackburn J.T., Padua D.A., Li L., Myers J.B. (2013). Effect of excessive contralateral trunk tilt on pitching biomechanics and performance in high school baseball pitchers. Am. J. Sports Med..

[43] Ferdinands R.E.D., Singh U. (2023). Investigating the biomechanical validity of the V-spine angle technique in cricket fast bowling. Int. J. Sports Sci. Coach..

[44] King M.A., Worthington P.J., Ranson C.A. (2016). Does maximising ball speed in cricket fast bowling necessitate higher ground reaction forces?. J. Sports Sci..

[45] Shapiro M.B., Aruin A.S., Latash M.L. (1995). Velocity-dependent activation of postural muscles in a simple two-joint synergy. Hum. Mov. Sci..

[46] Yu J., Ackland D.C., Pandy M.G. (2011). Shoulder muscle function depends on elbow joint position: An illustration of dynamic coupling in the upper limb. J. Biomech..

[47] Yoshii Y., Yuine H., Kazuki O., Tung W.-L., Ishii T. (2015). Measurement of wrist flexion and extension torques in different forearm positions. BioMed. Eng. Online.

[48] Beringer C.R., Mansouri M., Fisher L.E., Collinger J.L., Munin M.C., Boninger M.L., Gaunt R.A. (2020). The effect of wrist posture on extrinsic finger muscle activity during single joint movements. Sci. Rep..

[49] Fuss F.K., Doljin B., Ferdinands R.E.D. (2023). The biomechanics of converting torque into spin rate in spin bowlers analysed with a smart cricket ball. Ind. J. Orthop..

[50] Worthington P.J., King M.A., Ranson C.A. (2013). Relationships between fast bowling technique and ball release speed in cricket. J. Appl. Biomech..

[51] Fuss F.K., Doljin B., Ferdinands R.E.D. (2024). Calculation of the Point of Application (Centre of Pressure) of Force and Torque Imparted on a Spherical Object from Gyroscope Sensor Data, Using Sports Balls as Practical Examples. Sensors.

[52] Felton P.J., Yeadon M.R., King M.A. (2020). Optimising the front foot contact phase of the cricket fast bowling action. J. Sports Sci..

[53] Middleton K.J., Wells D.J., Foster D.H., Alderson J.A. (2018). Proximal cueing to reduce elbow extension levels in suspect spin bowlers: A case study. Int. J. Sports Sci. Coach..

[54] Galloway J.C., Koshland G.F. (2002). General coordination of shoulder, elbow and wrist dynamics during multi-joint arm movements. Exp. Brain Res..

[55] Schoenfeld B. (2002). Accentuating muscular development through active insufficiency and passive tension. Strength Cond. J..

[56] Escamilla R., Fleisig G., Barrentine S., Andrews J., Moorman C. (2002). Baseball: Kinematic and Kinetic comparisons between American and Korean professional baseball pitchers. Sports Biomech..

[57] Zajac F.E. (1993). Muscle coordination of movement: A perspective. J. Biomech..

[58] Ikeda K., Kaneoka K., Matsunaga N., Ikumi A., Yamazaki M., Yoshii Y. (2025). Effects of forearm rotation on wrist flexor and extensor muscle activities. J. Orthop. Surg. Res..

[59] Forman D.A., Forman G.N., Avila-Mireles E.J., Mugnosso M., Zenzeri J., Murphy B., Holmes M.W. (2020). Characterizing forearm muscle activity in young adults during dynamic wrist flexion–extension movement using a wrist robot. J. Biomech..

[60] Leech K.A., Roemmich R.T., Gordon J., Reisman D.S., Cherry-Allen K.M. (2022). Updates in Motor Learning: Implications for Physical Therapist Practice and Education. Phys. Ther..

[61] Sarpeshkar V., Mann D.L., Spratford W., Abernethy B. (2017). The influence of ball-swing on the timing and coordination of a natural interceptive task. Hum. Mov. Sci..

[62] Fuss F.K., Doljin B., Ferdinands R.E.D. (2018). Effect on Bowling Performance Parameters When Intentionally Increasing the Spin Rate, Analysed with a Smart Cricket Ball. Proceedings.

[63] De Luca C.J. (1997). The use of surface electromyography in biomechanics. J. Appl. Biomech..

[64] Kookaburra (2024). Smart Ball FAQs. https://www.kookaburrasport.com.au/cricket/team-kookaburra/innovation/.

[65] Kumar A., Espinosa H.G., Worsey M., Thiel D.V. (2020). Spin rate measurements in cricket bowling using magnetometers. Proceedings.

